# Glutathionylation of dengue and Zika NS5 proteins affects guanylyltransferase and RNA dependent RNA polymerase activities

**DOI:** 10.1371/journal.pone.0193133

**Published:** 2018-02-22

**Authors:** Chonticha Saisawang, Atichat Kuadkitkan, Prasert Auewarakul, Duncan R. Smith, Albert J. Ketterman

**Affiliations:** 1 Institute of Molecular Biosciences, Mahidol University, Salaya Campus, Salaya, Thailand; 2 Department of Microbiology, Faculty of Medicine Siriraj Hospital, Mahidol University, Bangkok, Thailand; Institut Pasteur of Shanghai Chinese Academy of Sciences, CHINA

## Abstract

It has been estimated for dengue infection that the global population at risk is 3.5 billion people, which makes dengue an important public health problem. The causative agents of dengue are dengue viruses. For dengue virus replication, the dengue virus NS5 protein is of special importance as it has several enzyme activities important for viral replication. Previous reports of phosphorylation and SUMOylation of dengue NS5 have shown these protein modifications have important consequences for NS5 functions. In this report we identify glutathionylation, another reversible post translation modification that impacts on NS5 enzyme activity. Using dengue virus infected cells we employed specific antibodies and mass spectrometry to identify 3 cysteine residues of NS5 protein as being glutathionylated. Glutathionylation is a post translational protein modification where glutathione is covalently attached to a cysteine residue. We showed glutathionylation occurs on 3 conserved cysteine residues of dengue NS5. Then we generated two flavivirus recombinant full length proteins, dengue NS5 and Zika NS5, to characterize two of the NS5 enzyme activities, namely, guanylyltransferase and RNA-dependent RNA polymerase activities. We show glutathionylation of dengue and Zika NS5 affects enzyme activities of the two flavivirus proteins. The data suggests that glutathionylation is a general feature of the flavivirus NS5 protein and the modification has the potential to modulate several of the NS5 enzyme functions.

## Introduction

It has been estimated for dengue infection that there is a population at risk of 3.5 billion people, this makes dengue one of the most important public health problems in most tropical and sub-tropical countries [1]. The meta study by Bhatt et al. further estimated that dengue may actually infect 390 million people per year of which some 96 million are symptomatic [1]. The causative agent of dengue, the dengue viruses (DENV) are transmitted to humans by the bite of infected female mosquitoes belonging to the Aedes family, most commonly *Aedes aegypti* and *Aedes albopictus* [2]. Unfortunately, *Ae*. *albopictus* is one of the world’s most invasive species [3–6].

The dengue viruses are classified in the family *Flaviviridae*, genus *Flavivirus*, and species *Dengue virus*, and comprise four antigenically distinct viruses termed dengue serotypes 1, 2, 3 and 4. Structurally, the mature DENV is an icosahedral, enveloped virus of approximately 50 nm and consists of three proteins, membrane (M), capsid (C) and envelope (E) and a single stranded, positive sense RNA genome of approximately 11k bases. The virus has a significant lipid component, and approximately 17% of the virion by weight is lipid [7] which forms a lipid bilayer between the nucleocapsid core and the E/M outer shell [8]. The RNA genome has a type 1 cap structure which mimics host mRNA, but no poly (A) tail. The genome encodes three structural proteins, with the membrane protein being encoded as a precursor form (preM) and seven non-structural proteins (NS1, NS2A, NS2B, NS3, NS4A, NS4B and NS5) which direct RNA replication.

In primary infections DENV is believed to enter into cells through clathrin coated pits by receptor mediated endocytosis [9, 10] after binding to a protein receptor or receptor complex expressed on the cell surface, although alternate pathways, particularly in mammalian cells have been proposed [11, 12]. In mosquito cells, entry of the virus is also believed to occur by receptor mediated endocytosis through clathrin coated pits. In clathrin mediated entry, the virus particle is inside an endocytic vesicle, and release of the nucleocapsid to the cytoplasm occurs after fusion of the E protein with the vesicle membrane under conditions of low pH. Translation of the dengue genomic RNA results in a single polypeptide which is cleaved into the three structural and seven non-structural proteins which serve to form the replication complex [13, 14]. Studies have shown that the replication complex is found in close association with the ER and other membranous structures [15].

While all of the non-structural proteins are important for dengue virus replication, the dengue virus NS5 protein is of special importance as it has several enzyme activities important for viral replication, namely, guanylyltransferase (GTase), RNA guanine-N7-methyltransferase (N7MTase), nucleoside 2’-O-methyltransferase (2’OMTase) and RNA-dependent RNA polymerase (RdRp) activities [16]. For an understanding of the flavivirus RdRp we direct readers to a recent review (see [17]). The guanylyltransferase and RNA methyltransferase activities generate a type 1 cap structure (^m7^GpppN_2’OMe_-RNA) on the 5’ end of the viral RNA [16]. This is the same type of cap structure found on eukaryotic mRNA and allows the viral RNA to evade the foreign RNA defense mechanisms of the host cell. NS5 GTase catalyzes a two-step reaction using GTP to produce a covalent GMP-enzyme intermediate. The second step of the reaction transfers the GMP to the diphosphate end of the RNA transcript to produce the GpppN structure. The guanosine base is methylated by N7MTase activity and the ribose of the adjacent nucleotide is methylated by 2’OMTase activity to generate the final RNA type 1 cap. In higher eukaryotes the type 1 cap plays many essential roles including nuclear export, initiation of mRNA translation, designator of self RNA and roles in innate immunity [18]. Viral RNA without the 5’ cap would thus be recognized as non-self in host cells and trigger an innate immune response. Therefore viruses have developed many diverse strategies to cap their RNA [18]. The GTase activity of NS5 is therefore catalysing an essential step in the production of viable viral RNA. Although NS5 has been shown to be a true GTase little is known of the amino acid residues involved in the catalytic mechanism [19]. Metazoan GTases have a consensus KxDG motif in which the ε-amino group of the active site lysine is covalently bound to GMP [20]. The flaviviruses do not have this motif. In addition, NS5 has also been shown conclusively by several groups employing multiple techniques to interact with at least 41 different host cell proteins [[21] and references therein]. The current literature suggests that these viral and host protein-protein interactions are important for viral propagation as the virus appears to shut down or hijack host cellular machinery. These events generate oxidative stress in the host cell [22, 23] which leads to glutathionylation of proteins in the cell [24–26].

Glutathionylation is a reversible posttranslational protein modification where glutathione is covalently attached to a cysteine residue. The reactive cysteine would have a low pK_a_, that is, be in a basic environment in the protein, to be nucleophilic and oxidized by addition of glutathione. The glutathionylation can occur through chemical mechanisms or be enzymatically driven by thiolase activity. Glutathionylation yields a net negative charge which impacts protein structure, function and subcellular localization [25, 27]. One function of glutathionylation is as a protein protective mechanism against oxidative damage where the damaged protein is usually degraded. Perhaps more importantly, the glutathionylation modification can also play a role analogous to phosphorylation which modulates the biological activity of the target protein, for example inhibition or activation of enzyme/protein function [25]. With the observation of dengue NS5 being glutathionylated in virus infected cells we sought to determine if the effects observed with dengue NS5 applied to other flaviviruses and so we tested a recombinant Zika NS5.

## Results and discussion

**Fig 1:**
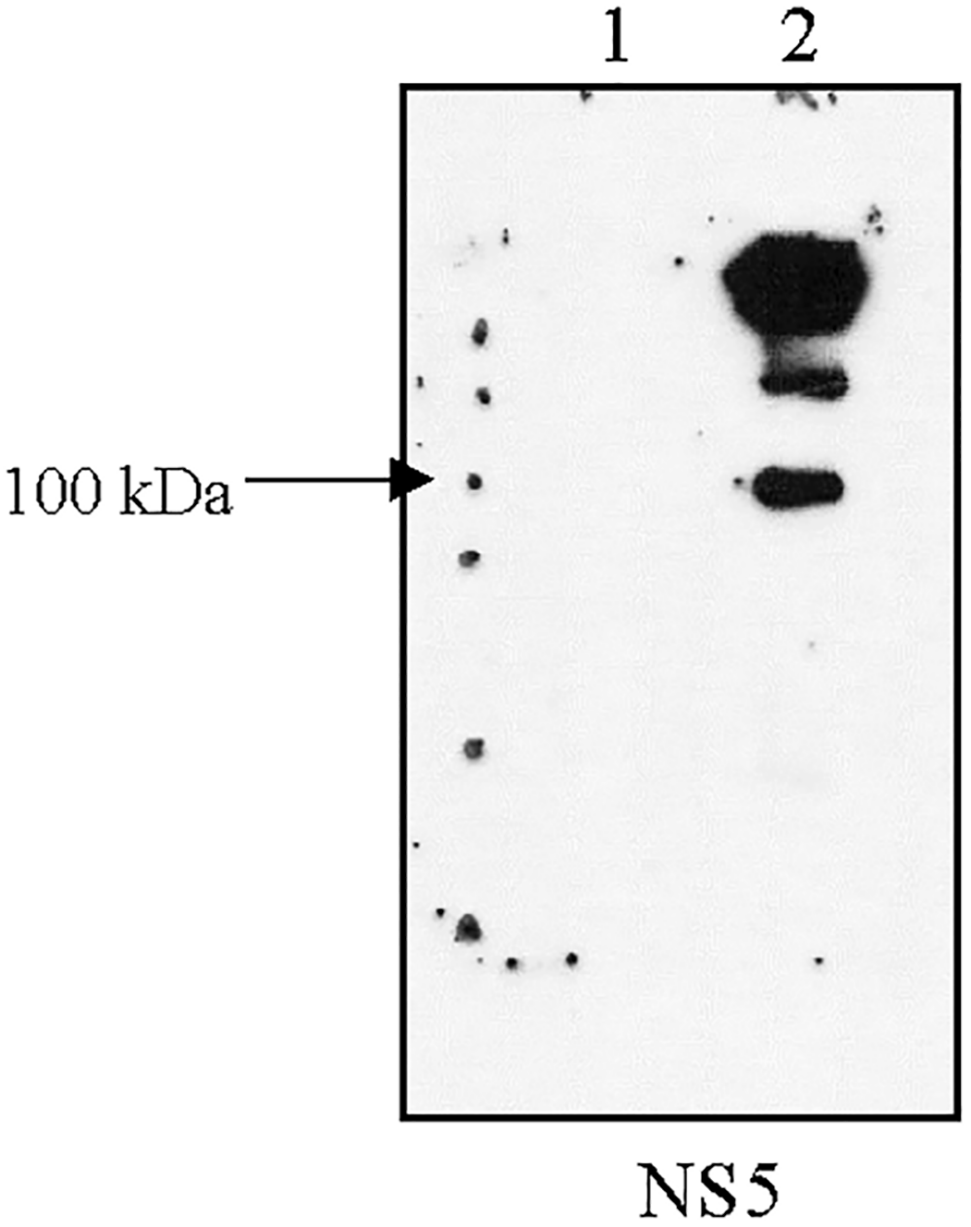
Immunoprecipitation result of the second method. The second method was to immunoprecipitate the dengue protein in cell lysate with specific dengue protein antibody and protein A/G beads and determine that the dengue protein was glutathionylated as shown by western blot detection with an anti-GSH antibody. The quantity of NS5 is not detectable in whole lysate. Lane 1 is DENV-infected cell lysate. Lane 2 is IP sample of NS5.

As stated above, the dengue virus genome encodes three structural proteins and seven non-structural proteins as a single open reading frame. The three structural proteins consist of capsid (C), membrane (M) and envelop (E) glycoproteins and the nonstructural proteins are the NS1, NS2A, NS2B, NS3, NS4A, NS4B and NS5 proteins. At this time there is no commercially available anti-NS2A antibody. Therefore only the remaining nine DENV proteins were screened for detection by antibody (S1 Fig). The signals of six dengue proteins were detected in the DENV-infected cells but not in mock-infected cells (S1 Fig). Antibodies against NS1, E and M did not give any signals. As 6 other dengue proteins were detected, this suggests that at one day post infection there was not sufficient quantity of these proteins in HEK293T/17 cells to be detected. We then performed immunoprecipitation with virus infected cells. The results suggested that NS1, NS3, NS4B and NS5 are modified by glutathionylation (S2 Fig). As the NS5 protein possesses several functions for viral propagation we decided to focus on this dengue protein. The reverse experiment, that is, immunoprecipitation of dengue NS5 protein with specific antibody followed by detection for glutathionylation by western blot using anti-GSH antibody confirmed NS5 is glutathionylated (Fig 1).

**Fig 2:**
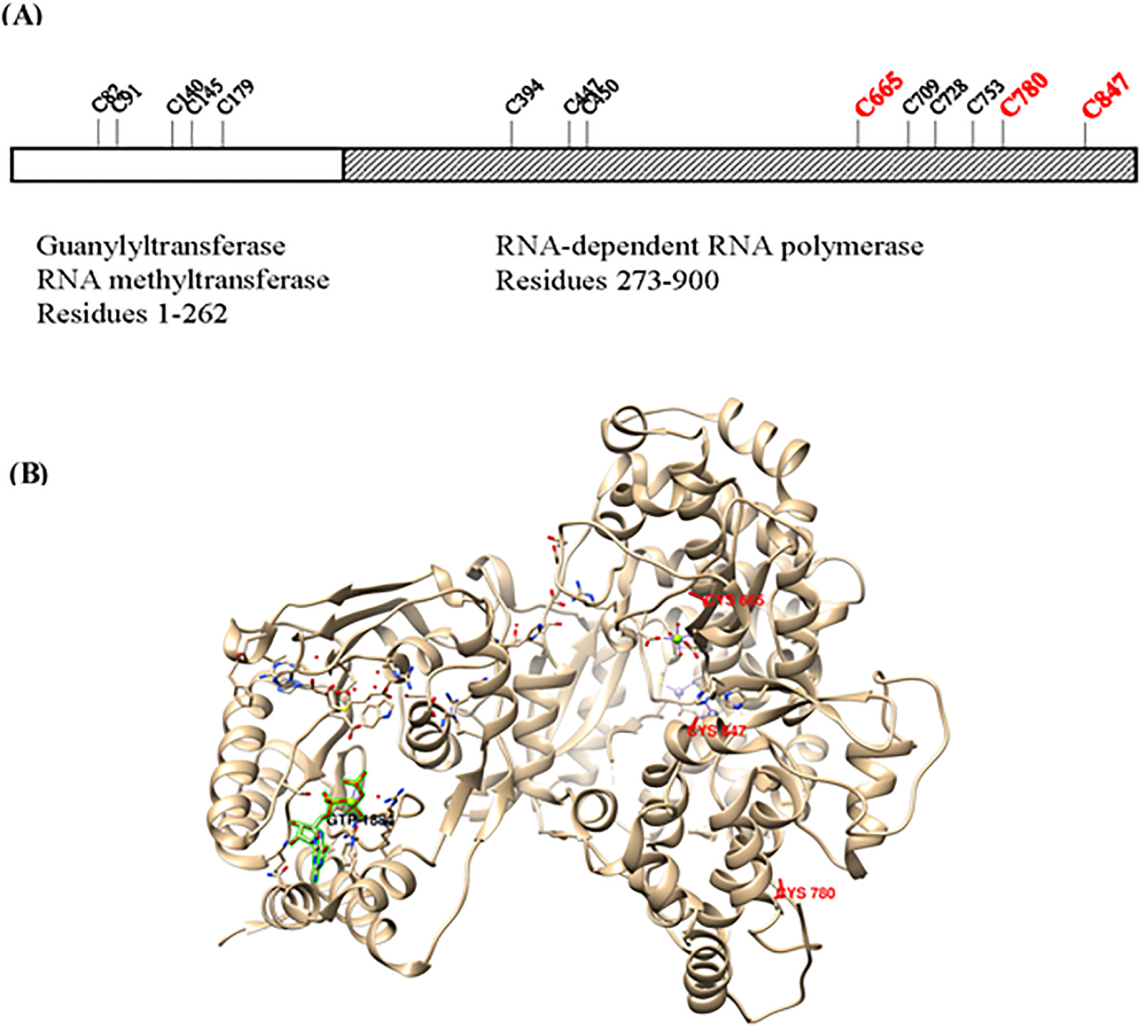
Schematic of DENV NS5 protein illustrating identified cysteine glutathionylation. **2A** Shown are the two domains of NS5 with respective activity and residue length underneath [28]. The 14 relative cysteine positions are shown with the identified glutathionylated cysteines shown in bold red. **2B** Shown is a ribbon representation of full length dengue NS5 (PDB ID 4V0R) with the identified glutathionylated cysteines shown in red and highlighted in green the GTP in the methyltransferase/guanylyltransferase domain.

DENV NS5 protein has a total of 14 cysteine residues that may be potential candidates for glutathionylation. We then resolved immunoprecipitants of lysates of DENV 2 virus infected HEK293T/17 cells on SDS-PAGE and excised the 103 kDa protein region for mass spectrometric analysis (S3 Fig). After several attempts that yielded 59% coverage of the full length NS5 we were able to identify 3 of the 14 cysteines in dengue NS5 as being glutathionylated. These 3 cysteines were Cys665, Cys780 and Cys847 with all 3 residues in the C-terminal half of the NS5 protein (Fig 2 and S1 Table). Many of the remaining cysteines were not detected and therefore later also may be found to be glutathionylated.

**Fig 3:**
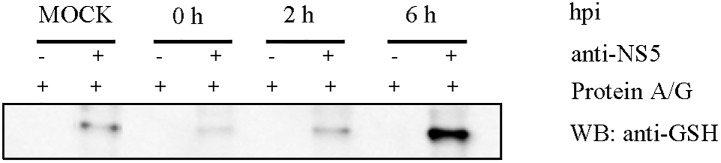
Time course of DENV NS5 glutathionylation in the virus infected cell. HEK293T/17 cells were mock infected and infected with DENV 2 for 2 h. The infected cells were harvested at 0, 2 and 6 hour post infection (hpi). The immunoprecipitates with DENV NS5 protein antibody were run on non-reduced SDS gel and western blot was performed using anti-GSH antibody.

We also performed a time course for the glutathionylation of dengue NS5 in virus infected HEK293T/17 cells (Fig 3). As can be observed in Fig 3 the NS5 antibody appeared to cross react with a host protein, however there appeared to be a substantial increase in NS5 glutathionylation after 6 hr. A Coomassie stained SDS-PAGE gel performed in parallel showed the NS5 protein band, which was confirmed to be NS5 by mass spectrometry, but the protein in the mock lane could not be detected. No protein band being detected precluded us from identifying the immunoreacting host protein. However the increased glutathionylation of NS5 correlates with reports of DENV viral RNA accumulation after a 6 hr lag period [29], which suggests the concomitant increases in viral proteins that we observe.

**Fig 4:**
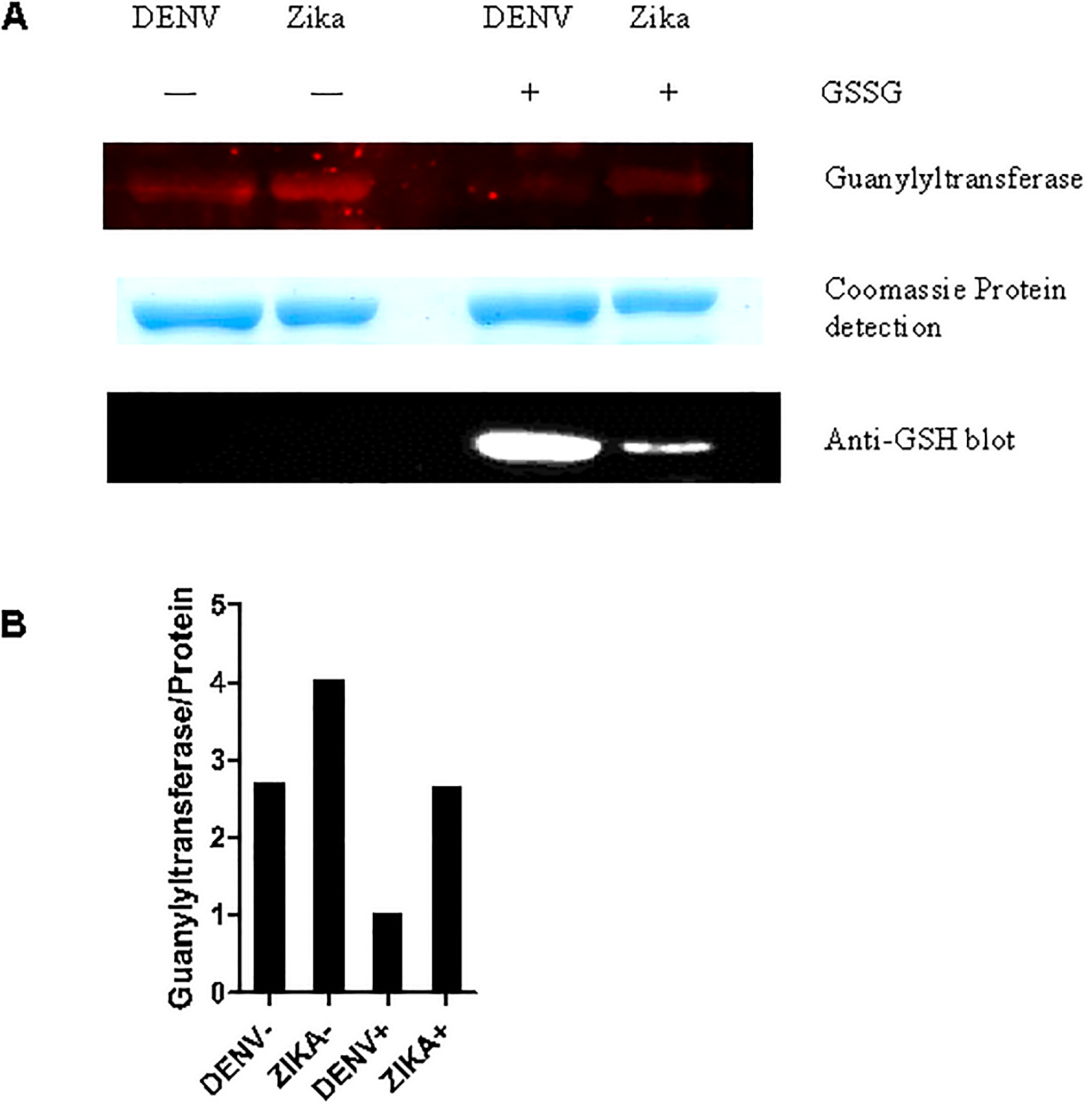
Glutathionylation effects on dengue and Zika NS5 guanylyltransferase activity. A) DENV and Zika NS5 protein were not treated and pre-treated with 5 mM GSSG for 10 min at RT. These proteins then were used to perform the guanylyltransferase activity assay. The samples were resolved on 10% SDS-PAGE. The extent of enzyme guanylylation activity was measured by GTP-Cy5 signal tracking using Azure^™^ cSeries. Gels were then stained with Coomassie Blue to normalize for protein loading. The bottom panel shows equivalent aliquots of the same samples used in a parallel gel that was transferred for Western blot and detection by anti-GSH antibody. B) The ratio of quantified guanylyltransferase bands to protein load of panel A is shown as a bar graph.

**Fig 5:**
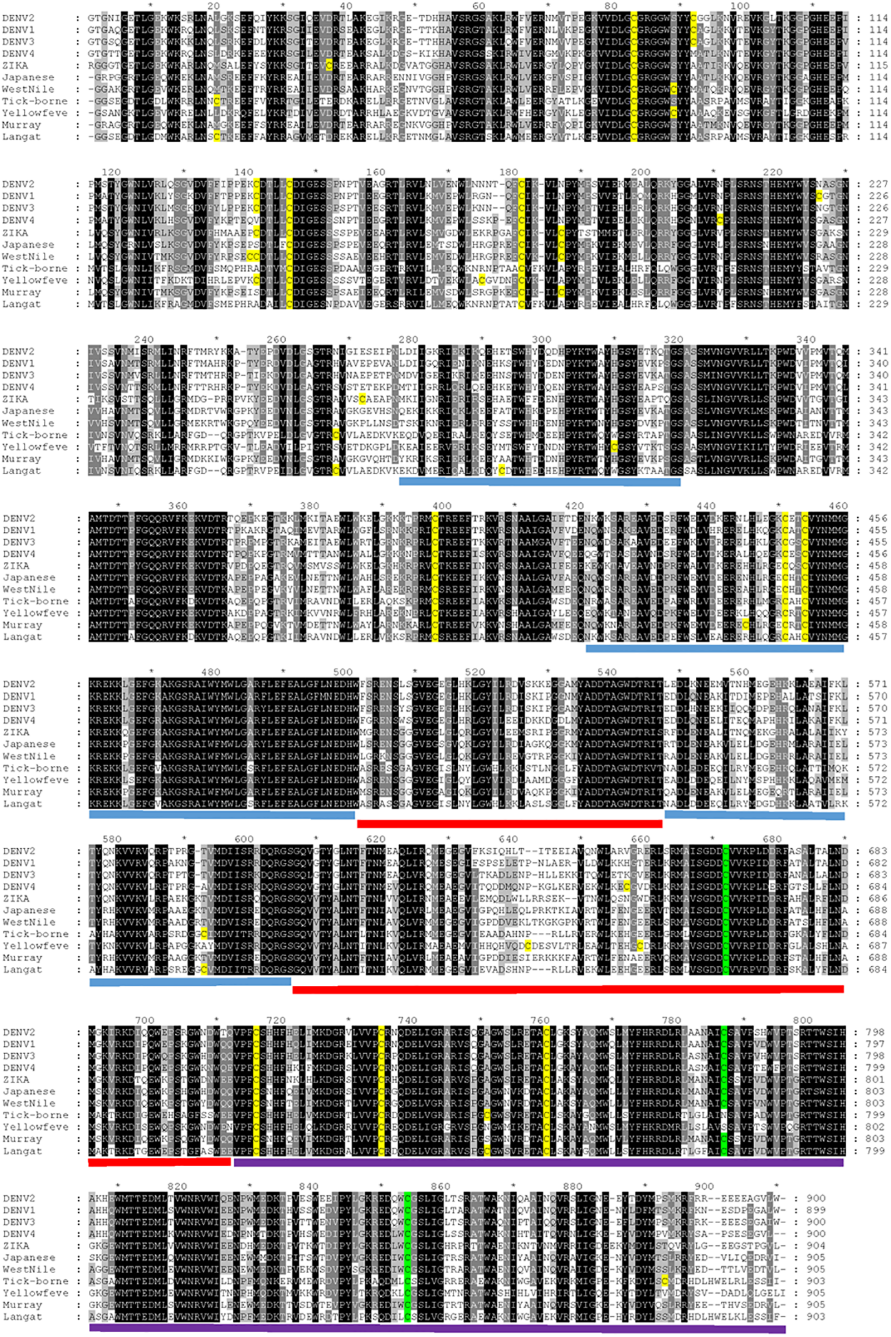
Amino acid alignment of flavivirus NS5 proteins. The sequences are nonstructural protein NS5 from Dengue virus 2 NP_739590.2, Dengue virus 1 NP_722465.1, Dengue virus 3 YP_001531176.2, Dengue virus 4 NP_740325.1, Zika virus AMR39834.1, Japanese encephalitis virus NP_775674.1, West Nile virus YP_001527887.1, Tick-borne encephalitis virus NP_775511.1, Yellow fever virus NP_776009.1, Murray Valley encephalitis virus NP_722539.1, Langat virus NP_740302.1. Cysteines are highlighted in yellow with the positions of the 3 glutathionylated cysteines identified in this study shown in green highlight in the alignment. Blue bars illustrate the residues of the finger subdomains, the red bars show the residues of the palm domain and the purple bars show the residues of the thumb domain [30]. Conservation of amino acid sequence is shown by 100 percent conserved as white on black, between 80 to 100 percent conserved as white on dark grey, between 60 to 80 percent conserved as black on light grey, and less than 60 percent conserved as black on white.

With the confirming results from the virus infected cells, we generated constructs for recombinant full length NS5 protein expression. As these 3 cysteines are highly conserved in flavivirus NS5, we also tested a second flavivirus recombinant NS5. The constructs generated included a dengue NS5 as well as a Zika virus NS5. The recombinant NS5 proteins were then studied for glutathionylation effects on GTase and RdRp activities. We observed under the assay conditions that the Zika NS5 showed more GTase activity than the dengue NS5 and the activity was affected less than the dengue NS5 activity after glutathionylation (Fig 4). With only 65% amino acid identity between the dengue 2 NS5 and Zika NS5 proteins (Fig 5) we suggest that the glutathionyl modification causes changes in the protein dynamics that is different in the two proteins and that impacts on the observed GTase activity.

**Fig 6:**
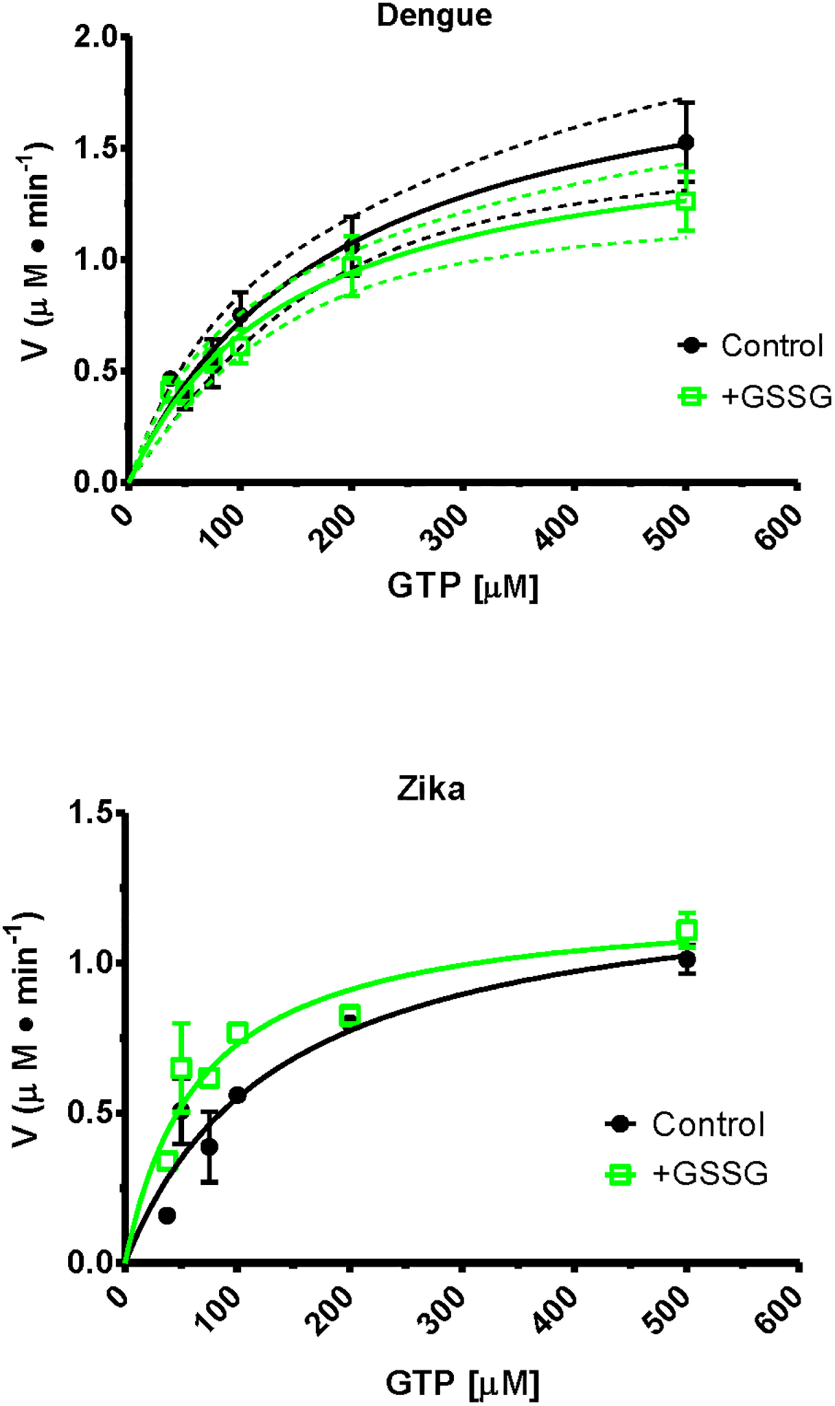
Plots of V versus S for dengue and Zika NS5 RdRp activity before and after glutathionylation with the kinetic parameter values generated by non-linear regression of this secondary plot. On the dengue plot the 95% confidence limits for the curves are shown by dotted lines. The plots were generated by GraphPad Prism version 5.04. See S4 Fig for an example of the primary plot.

To obtain kinetic constants for the RdRp assay it was performed as a time course for varying GTP concentrations and the slopes of the data plots (enzyme rates) were used on a secondary plot of V/S (for an example see S4 Fig). Replicate experiments yielded kinetic constants and the plots shown (Fig 6). The glutathionylated dengue NS5 had a V_max_ 1.633 ± 0.182 μM/min and a K_m_ 146.2 ± 37.3 μM. This compares to control V_max_ 2.094 ± 0.272 μM/min and K_m_ 189.8 ± 52.5 μM. In comparison the Zika NS5 is slightly different with the glutathionylated NS5 having a V_max_ 1.213 ± 0.107 μM/min and a K_m_ 66.34 ± 16.86 μM. This compares to Zika NS5 control V_max_ 1.306 ± 0.172 μM/min and K_m_ 137.2 ± 41.3 μM. Although lacking significance for the final kinetic parameter values, the trends of the kinetic data plots show differences for both dengue and Zika enzymes after glutathionylation (Fig 6). Each of the dengue curves lie outside the 95% confidence limits of the other curve but this is not the case for the Zika enzyme curves. However, the Zika NS5 appears to significantly increase affinity of binding for the GTP. This suggests the two enzymes RdRp activities are affected by glutathionylation but also behave in slightly different ways. This is interesting as it occurs for the flavivirus NS5 activities at higher concentrations of GTP. These concentrations, of 0.3 to 0.5 mM, are the physiological concentrations which are found in the cell [31]. As stated above, the 3 cysteines identified as being glutathionylated are highly conserved residues in flavivirus NS5 proteins (Fig 5). Two of the residues, Cys780 and Cys847, are in the thumb domain with Cys780 being proximal to the priming loop of residues 782–809 and Cys847 being one of four residues that coordinates one of the two zinc atoms found in NS5 proteins. That zinc atom was proposed to contribute to structural stability of the region as well as regulating thumb subdomain conformational changes that occur during the reaction pathway [30]. A recent structural study also showed that the thumb subdomain was more dynamic than the finger and palm subdomains with greater deuterium-exchange as well as higher crystallographic temperature factors [28]. The authors also suggested that cross talk between the N-terminus domain and the fingers subdomain contributed to various conformational ensembles for interaction with various viral proteins, RNA and host cofactors during the virus replication cycle [28]. Thus amino acid modifications in these regions may have significant influence on the protein’s structural dynamics. Therefore our identified cysteine modifications would impact structurally on several aspects of NS5 and these effects can be observed in changes for both guanylyltransferase activity and the RdRp activity. Reports of the N-terminal domain modifying/contributing to polymerase activity has been reported previously [32], and with the modified cysteines in the C-terminal RdRp affecting the activity of the N-terminal guanylyltransferase we suggest that the reverse communication also occurs.

Several reports suggest that virus induced oxidative stress may regulate host response through a protein post translational modification such as glutathionylation [29, 33]. For dengue NS5 specifically it has been reported that different extents of phosphorylation control cellular localization in the cytosol or nucleus, as well as the ability of NS5 to interact with NS3 and function in viral RNA replication [34, 35]. Recently dengue NS5 has also been reported to be modified by small ubiquitin-like modifier (SUMO), a second reversible posttranslational modification that also regulates a variety of cellular systems [36]. The SUMOylation of NS5 appeared to be important for viral replication, increasing stability of NS5 and suppressing STAT2 degradation which inhibits IFN function and thereby suppresses innate immunity. As previously stated, protein glutathionylation is a reversible posttranslational modification that also has been reported to modulate many cellular processes. In this report we have shown NS5 to be glutathionylated both for dengue as well as a second flavivirus Zika. The cysteine residues we found to be glutathionylated are highly conserved across multiple flaviviruses (Fig 5). This suggests that glutathionylation may be a general feature for flavivirus NS5. We also show that this modification occurs multiple times on the protein and can modulation NS5 enzyme activities. How these modifications affect the various NS5 functions in the cell remains to be addressed.

## Methods

### DENV 2 virus production in HEK293T/17 cells

Human Embryonic Kidney 293T/17 cells (HEK293T/17; ATCC Cat No. CRL-11268) were cultured at 37°C, 5% CO_2_ in Dulbecco’s modified Eagle’s medium (DMEM; Gibco, Invitrogen, Carlsbad, CA) supplemented with 10% heat-inactivated fetal bovine serum (FBS; Gibco, Invitrogen) and 100 units of penicillin and 100 μg streptomycin/ml. Cells were either mock-infected or infected with DENV 2 (strain 16681) at a multiplicity of infection (MOI) of 5. Viral absorption was allowed to proceed for 2 h at 37°C with constant agitation. After this period cells were washed 3 times with PBS to remove unabsorbed viruses. Fresh culture medium was added to the cells and cells were further incubated under standard conditions for 1 day.

### Time course of DENV 2 NS5 glutathionylation during viral infection

HEK293T/17 cells were seeded on 10 mm tissue culture plates at a density that allowed 80% confluence within 24 hours. The cells were infected with DENV 2 at MOI of 5 for 2 h. The virus was then removed and the cells were further incubated under standard conditions and harvested at 0, 2 and 6 h post infection (hpi). Cells were lysed and measured for protein concentration by Bradford method using BSA as standard. 1 mg of cell lysates were immunoprecipitated with 1 mg/ml of an anti-DENV 2 NS5 protein monoclonal antibody (GeneTex). The immunoprecipitates were used for 10% SDS-PAGE and western blot analysis and detected with an anti-glutathione (GSH) antibody (US Biological).

### Cell lysate preparation

Mock-infected and DENV 2 infected cell pellets were lysed in NP-40 lysis buffer (50 mM Tris, pH 8, 150 mM NaCl and 1% NP-40). Cell pellets were incubated on ice and vigorously vortexed every 10 min for 30 min total time. Cell pellets were centrifuged at 10,000g, 4°C for 10 min. The cell lysate supernatants were measured for protein concentration as described above.

### Antibodies

Anti-dengue virus serotype 1–4 monoclonal antibody from Thermo Fisher Scientific (MA1-27093) (against E-protein). Anti-dengue virus prM glycoprotein antibody [DM1] from Abcam (ab41473). Anti-Dengue Virus 2 antibody from Abcam (ab155042) (against Capsid protein). Dengue virus type 2 NS1 antibody from Thermo Fisher Scientific (PA5-32207). Dengue virus NS2B protein antibody from GeneTex (GTX124246). Dengue virus type 2 NS3 antibody from Thermo Fisher Scientific (PA5-32199). Dengue virus type 2 NS4A antibody from Thermo Fisher Scientific (PA5-32197). Dengue virus type 2 NS4B antibody from Thermo Fisher Scientific (PA5-32198). Dengue virus type 2 NS5 protein antibody from GeneTex (GTX629446). Mouse anti-glutathionylation antibody was from USBiological (G8123).

### Immunoprecipitation (IP)

500 μg of protein cell lysates of mock and DENV 2 infected cells were incubated with 1 mg/ml of specific primary antibody with gentle rocking for 3 h at 4°C. Protein A/G Plus-Agarose (Santa Cruz) was subsequently added to precipitate protein complexes by further incubation with gentle rocking overnight at 4°C. The precipitates were collected by centrifugation at 6000g for 5 min and the supernatants were discarded. The pellets were washed 3 times with PBS buffer and resuspended in 30 μl of SDS sample loading buffer, and proteins were heated to 100°C for 5 min, followed by centrifugation at 10,000g for 5 min before applying the samples to 10 SDS-PAGE for western blot analysis.

The IP was performed in 2 different ways. The first method was to incubate cell lysates with an anti-glutathione (GSH) primary antibody to pull down all glutathionylated proteins in the cell lysate and then probed with each anti-dengue virus antibody by western blot. The second method was for reverse confirmation by immunoprecipitating the dengue virus protein with the specific primary antibody and then probing with an anti-GSH antibody.

### Western blot analysis

The protein samples were resolved under 10% SDS-PAGE. Proteins were transferred onto nitrocellulose membranes. The membranes were blocked with 5% skim milk in TBS-T at room temperature for 1 h and subsequently incubated overnight with an appropriate primary antibody, followed by incubation with an appropriate secondary antibody for 2 h at RT. The signals were developed using either ECL Plus Western Blotting analysis kit for HRP-conjugate or Lumi-Phos^™^ WB for AP-conjugate. The protein samples were run under non-reducing gel conditions (no DTT) if the anti-GSH primary antibody was used for analysis.

### Analysis of protein glutathionylation by western blot

5 mM oxidized glutathione (GSSG) was used to induce glutathionylation. Purified protein samples were used for 10% SDS-PAGE under non-reducing conditions (no DTT) and transferred to nitrocellulose membrane. Blots were incubated with primary anti-glutathione antibody (1:1000) at 4°C overnight and then incubated with an appropriate secondary antibody. The signals were detected with enhanced chemiluminescent detection reagents (ECL).

### Immunoprecipitation of DENV NS5 protein from virus infected cells for cysteine glutathionylation site identification by mass spectrometry

HEK293T/17 cells were seeded on 10 mm tissue culture plates at a density that allowed 80% confluence within 24 hours. The cells were infected with DENV 2 at MOI of 5 for 2 h. The virus was then removed and the cells were further incubated under standard conditions. Mock infected cells were generated in parallel. The cell lysate supernatants were prepared and measured for protein concentration as described above. Cell lysate was then treated with 5 mM GSSG at room temperature for 10 min. A non-treated sample was used as control. 1 mg of non-treated and treated cell lysates were used for IP with anti-DENV 2 NS5 protein antibody. The IP samples were then used for 10% SDS-PAGE followed by staining with Coomassie Blue. The protein bands of about 103 kDa were excised and sent for LC/MS/MS analysis. The LC/MS/MS was performed by Dr. Sze Siu Kwan at the NTU Mass Spec core facility at the School of Biological Sciences, Nanyang Technological University, Singapore.

### Construction of recombinant full length NS5 from dengue and Zika virus

RNA was extracted from DENV 2 virus stock using Illustra RNA spin mini RNA Isolation kit (GE Healthcare). The first-strand cDNA was synthesized from 0.1 μg of RNA using ImProm-II ^™^ reverse transcriptase (Promega). The cDNA primer was designed from the 3′UTR region of the virus sequence. The nucleotide sequences (2,700 NTs) of the DENV NS5 was amplified from cDNA template using virus specific primers. For Zika NS5 sequence (2,712 NTs) was amplified from sequence of Cambodia isolate FSS13025, Genbank number AFD30972.1.

The forward and reverse primers of both NS5 genes were designed to contain *Xho*I and *kpn*I restriction sites. The purified PCR products were digested with *Xho*I and *kpn*I and ligated into our engineered vector derived from pET21d. The recombinant plasmids were transformed into *E*.*coli* DH5α. The candidate clones were first screened by a rapid size screening method before final verification by DNA sequencing.

### Full length dengue and Zika NS5 protein expression and purification

The NS5 clones were extracted and retransformed into *E*. *coli* BL21-CodonPlus^®^ (DE3)-RIL competent cells for protein expression. The cells were cultured in terrific broth supplemented with 100 μg/ml ampicillin and 34 μg/ml chloramphenicol at 37°C until the cell density reached 0.5 at OD_600nm_. Protein expression was induced with 0.2 mM isopropyl-β-D-thiogalactopyranoside (IPTG) at 18°C for 16 h. The cells were subsequently centrifuged and kept at -20°C until use.

The cells were lysed in HisTrap (GE Healthcare) buffer, 20 mM Tris-HCl pH 7.5, 0.5 M NaCl, 0.2 M arginine and 0.2 M glutamic acid containing 0.5 mg/ml lysozyme and 1 mM DTT. The lysate was centrifuged at 10,000g for 30 min at 4°C, then applied to a HisTrap column. The column was washed with 10 column volumes of HisTrap buffer. Then the column was loaded with 1 column volume of HisTrap buffer containing 600 mg Precission protease to cleave the MBP-HIS tag at room temperature for 30 min. The HisTrap column was washed with 2 column volumes of binding buffer and this wash was applied to a maltose binding protein (MBP) affinity column. This bound cleaved tag that co-eluted with the NS5 protein from the HisTrap column. The NS5 protein flowed through the MBP column. Final yield of NS5 protein was about 2 mg per liter of expression culture. The purity of the protein was evaluated by SDS-PAGE.

### Guanylyltransferase (GTase) activity assay

8-[(6-Amino)hexyl]-amino-GTP-Cy5 (Jena Bioscience, Germany) is a GTP analog labeled with the fluorescent dye Cy5 and was used as a nucleotide substrate in the assay adapted from [37]. The standard reaction was set up as 5 μM purified enzyme, 5 mM Tris-HCl, pH 7.5, 3 μM GTP-Cy5, 0.5 mM MgCl_2_ and 0.1% NP-40, 0.2 M arginine and 0.2 M glutamic acid in 10 μl volume and incubated at 37°C for 2 h. Then 4X SDS-PAGE loading buffer was added to the reactions which were then denatured at 100°C for 5 min. The reactions were resolved on 10% SDS-PAGE. The extent of NS5 protein guanylylation was quantified by measuring the fluorescent signal intensity using an Azure^™^ cSeries. Then the same gel was stained with Coomassie blue and scanned with the same Gel Imaging system to normalize for protein loading.

*In vitro* glutathionylation of purified NS5 protein was performed by incubating protein with GSSG at room temperature for 10 min before performing the guanylyltransferase assay.

### RNA-dependent RNA polymerase (RdRp) activity assay

The RdRp activity assay was performed essentially as previously published [38]. In general, the assay was in 25 μl of 25 mM Tris-HCl pH 7.5 containing 2 μM RdRp, 2.5 mM MgCl_2_, 0.1 μM poly (rC) template, 1 mM GTP, 20 U/ml RNase inhibitor and 3 μM thermostable pyrophosphatase (PPase). The reaction mixture was incubated at 30°C for the desired time period, then heated to 75°C for 10 min (to convert pyrophosphate to inorganic phosphate by PPase and denature RdRp). Then 10 μl of reaction mixture was mixed with 200 μl malachite green-molybdate reagent and incubated at room temperature for 5 min before measuring absorbance at 650 nm. To determine kinetic parameters the GTP concentration was varied in the mixture as well as observing a time course for linear reaction rates. The reaction mixture without RdRp served as blank. The standard curve range was from 10 μM to 250 μM.

*In vitro* glutathionylation of purified NS5 protein was performed by incubating protein with GSSG at room temperature for 10 min before performing the RdRp activity assay.

## Supporting information

S1 Fig Western blot detection of each dengue protein in HEK293T/17 infected cells. Lane 1 is mock-infected cell lysate. Lane 2 is DENV-infected cell lysate. 50 μg of protein samples were loaded onto SDS-PAGE. Precision Plus ProteinTM standards was used (BioRad). Each specific antibody against Dengue proteins were employed for western blot detection as described.
(PDF)

S2 Fig Immunoprecipitation results of the first method. The first method was to immunoprecipitate the glutathionylated proteins in cell lysate with protein A/G beads and determine that dengue proteins were immunoprecipitated by detection with specific anti-dengue antibodies. Lane 1 is DENV-infected cell lysate. Lane 2 is IP sample.
(PDF)

S3 Fig SDS gels of DENV 2 infected cell lysates used for mass spectrometry determination of NS5 glutathionylation sites. The first lane is molecular weight marker and the other lanes are DENV 2 infected HEK293T/17 cell lysates. The gel slices of about 103 kDa were excised and sent for LC/MS/MS analysis. The LC/MS/MS was performed by Dr. Sze Siu Kwan at the NTU Mass Spec core facility at the School of Biological Sciences, Nanyang Technological University, Singapore.
(PDF)

S4 Fig A typical experimental determination of kinetic parameters for RdRp activity of NS5. A) An example of the time course for GTP hydrolysis to determine steady state kinetics. B) The enzyme rates (slopes) from the time course plots versus GTP concentrations. This is a V versus S plot with the kinetic parameter values generated by non-linear regression of this secondary plot. The plots were generated by GraphPad Prism version 5.04.
(PDF)

S1 Table Dengue NS5 peptides identified by mass spectrometry as containing the glutathionylated cysteine. The glutathionylated cysteine is shown in red with the residue number given in superscript. LC/MS/MS was performed by Dr. Sze Siu Kwan at the NTU Mass Spec core facility at the School of Biological Sciences, Nanyang Technological University, Singapore.
(DOCX)

## Abbreviations

DENV: dengue virus(es)
GTase: guanylyltransferase
NS5: nonstructural protein 5
RdRp: RNA-dependent RNA polymerase
