# Supplementary material for: Glutathionylation of dengue and Zika NS5 proteins affects guanylyltransferase and RNA dependent RNA polymerase activities
Source: PLoS One. 2018 Feb 22;13(2):e0193133. doi: 10.1371/journal.pone.0193133 (PMC5823458; doi:10.1371/journal.pone.0193133)
Supplement: S1 Fig — Lane 1 is mock-infected cell lysate. Lane 2 is DENV-infected cell lysate. 50 μg of protein samples were loaded onto SDS-PAGE. Precision Plus ProteinTM standards was used (BioRad). Each specific antibody against Dengue proteins were employed for western blot detection as described. (PDF) [file pone.0193133.s001.pdf]

S1 Fig

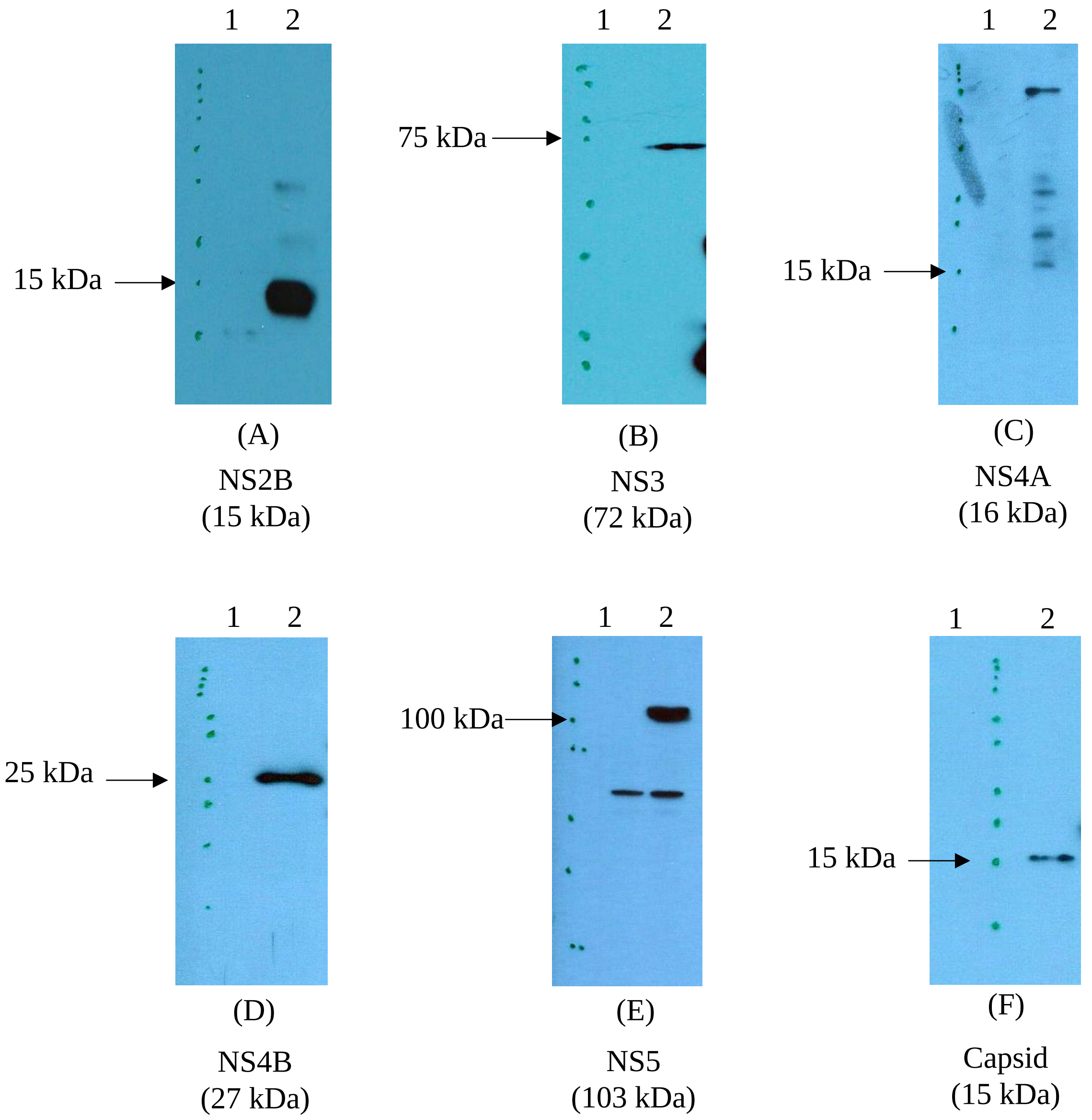

S1 Fig. Western blot detection of each dengue protein in HEK293T/17 infected cells. Lane 1 is mock-infected cell lysate. Lane 2 is DENV-infected cell lysate. 50 µg of protein samples were loaded onto SDS-PAGE. Precision Plus Protein^{TM} standards was used (BioRad). Each specific antibody against Dengue proteins were employed for western blot detection as described.
