# Supplementary material for: Glutathionylation of dengue and Zika NS5 proteins affects guanylyltransferase and RNA dependent RNA polymerase activities
Source: PLoS One. 2018 Feb 22;13(2):e0193133. doi: 10.1371/journal.pone.0193133 (PMC5823458; doi:10.1371/journal.pone.0193133)
Supplement: S2 Fig — The first method was to immunoprecipitate the glutathionylated proteins in cell lysate with protein A/G beads and determine that dengue proteins were immunoprecipitated by detection with specific anti-dengue antibodies. Lane 1 is DENV-infected cell lysate. Lane 2 is IP sample. (PDF) [file pone.0193133.s002.pdf]

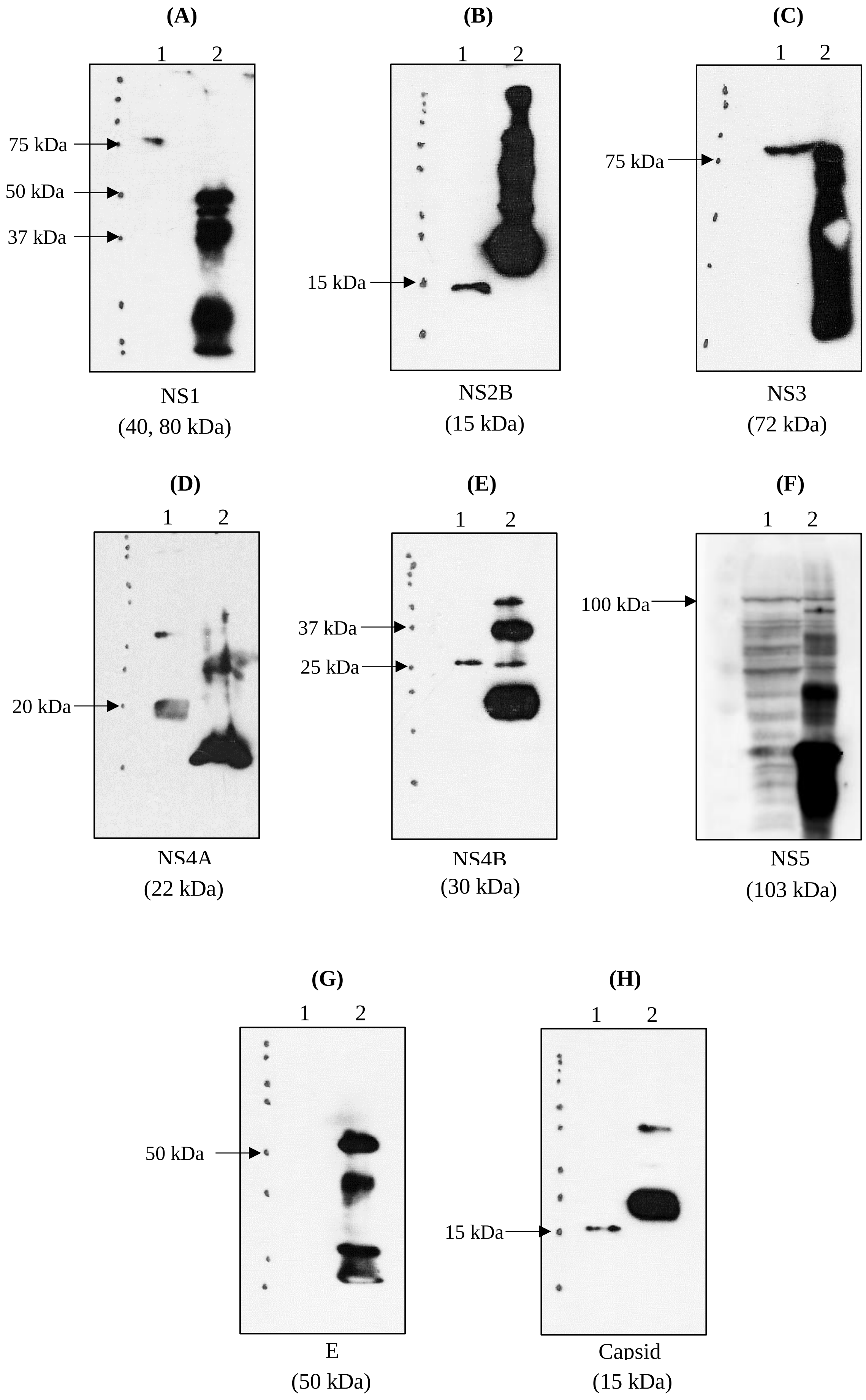
(A)
1 2
75 kDa
50 kDa
37 kDa
NS1
(40, 80 kDa)
(B)
1 2
15 kDa
NS2B
(15 kDa)
(C)
1 2
75 kDa
NS3
(72 kDa)
(D)
1 2
20 kDa
NS4A
(22 kDa)
(E)
1 2
37 kDa
25 kDa
NS4B
(30 kDa)
(F)
1 2
100 kDa
NS5
(103 kDa)
(G)
1 2
50 kDa
E
(50 kDa)
(H)
1 2
15 kDa
Capsid
(15 kDa)

**S2 Fig. Immunoprecipitation results of the first method.** The first method was to immunoprecipitate the glutathionylated proteins in cell lysate with protein A/G beads and determine that dengue proteins were immunoprecipitated by detection with specific anti-dengue antibodies. Lane 1 is DENV-infected cell lysate. Lane 2 is IP sample.
