# Supplementary material for: Glutathionylation of dengue and Zika NS5 proteins affects guanylyltransferase and RNA dependent RNA polymerase activities
Source: PLoS One. 2018 Feb 22;13(2):e0193133. doi: 10.1371/journal.pone.0193133 (PMC5823458; doi:10.1371/journal.pone.0193133)
Supplement: S3 Fig — The first lane is molecular weight marker and the other lanes are DENV 2 infected HEK293T/17 cell lysates. The gel slices of about 103 kDa were excised and sent for LC/MS/MS analysis. The LC/MS/MS was performed by Dr. Sze Siu Kwan at the NTU Mass Spec core facility at the School of Biological Sciences, Nanyang Technological University, Singapore. (PDF) [file pone.0193133.s003.pdf]

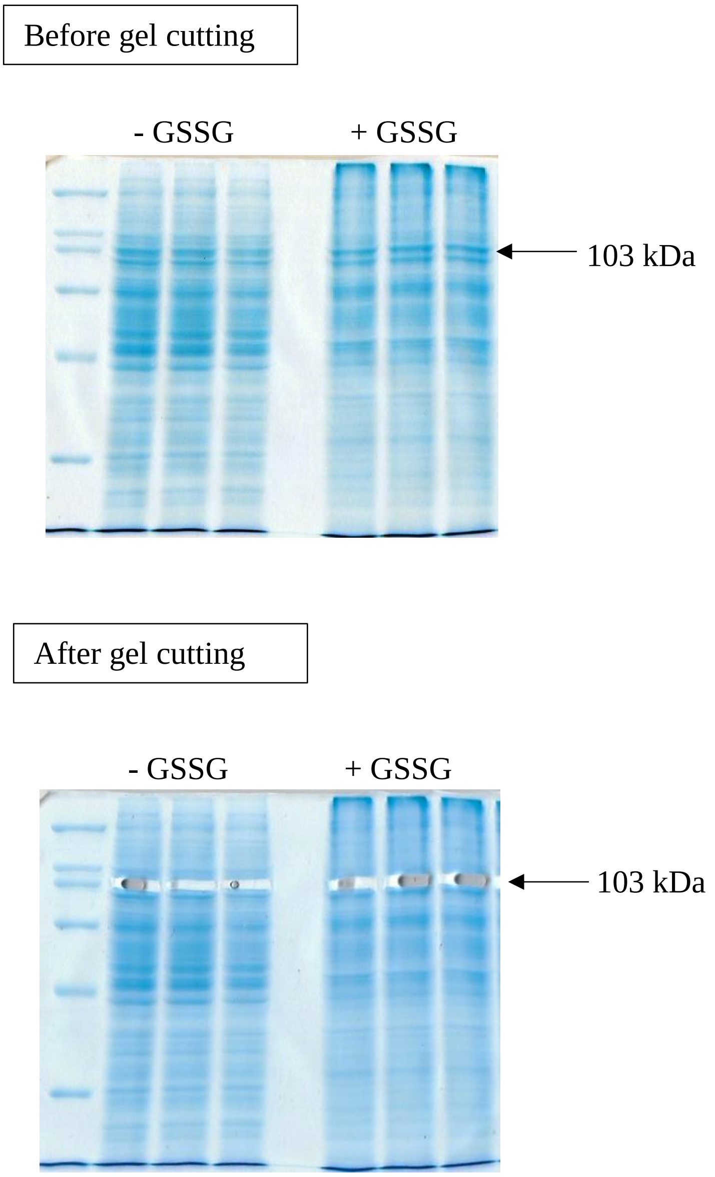


**S3 Fig. SDS gels of DENV 2 infected cell lysates used for mass spectrometry determination of NS5 glutathionylation sites.** The first lane is molecular weight marker and the other lanes are DENV 2 infected HEK293T/17 cell lysates. The gel slices of about 103 kDa were excised and sent for LC/MS/MS analysis. The LC/MS/MS was performed by Dr. Sze Siu Kwan at the NTU Mass Spec core facility at the School of Biological Sciences, Nanyang Technological University, Singapore.
