# Supplementary material for: Glutathionylation of dengue and Zika NS5 proteins affects guanylyltransferase and RNA dependent RNA polymerase activities
Source: PLoS One. 2018 Feb 22;13(2):e0193133. doi: 10.1371/journal.pone.0193133 (PMC5823458; doi:10.1371/journal.pone.0193133)
Supplement: S4 Fig — A) An example of the time course for GTP hydrolysis to determine steady state kinetics. B) The enzyme rates (slopes) from the time course plots versus GTP concentrations. This is a V versus S plot with the kinetic parameter values generated by non-linear regression of this secondary plot. The plots were generated by GraphPad Prism version 5.04. (PDF) [file pone.0193133.s004.pdf]

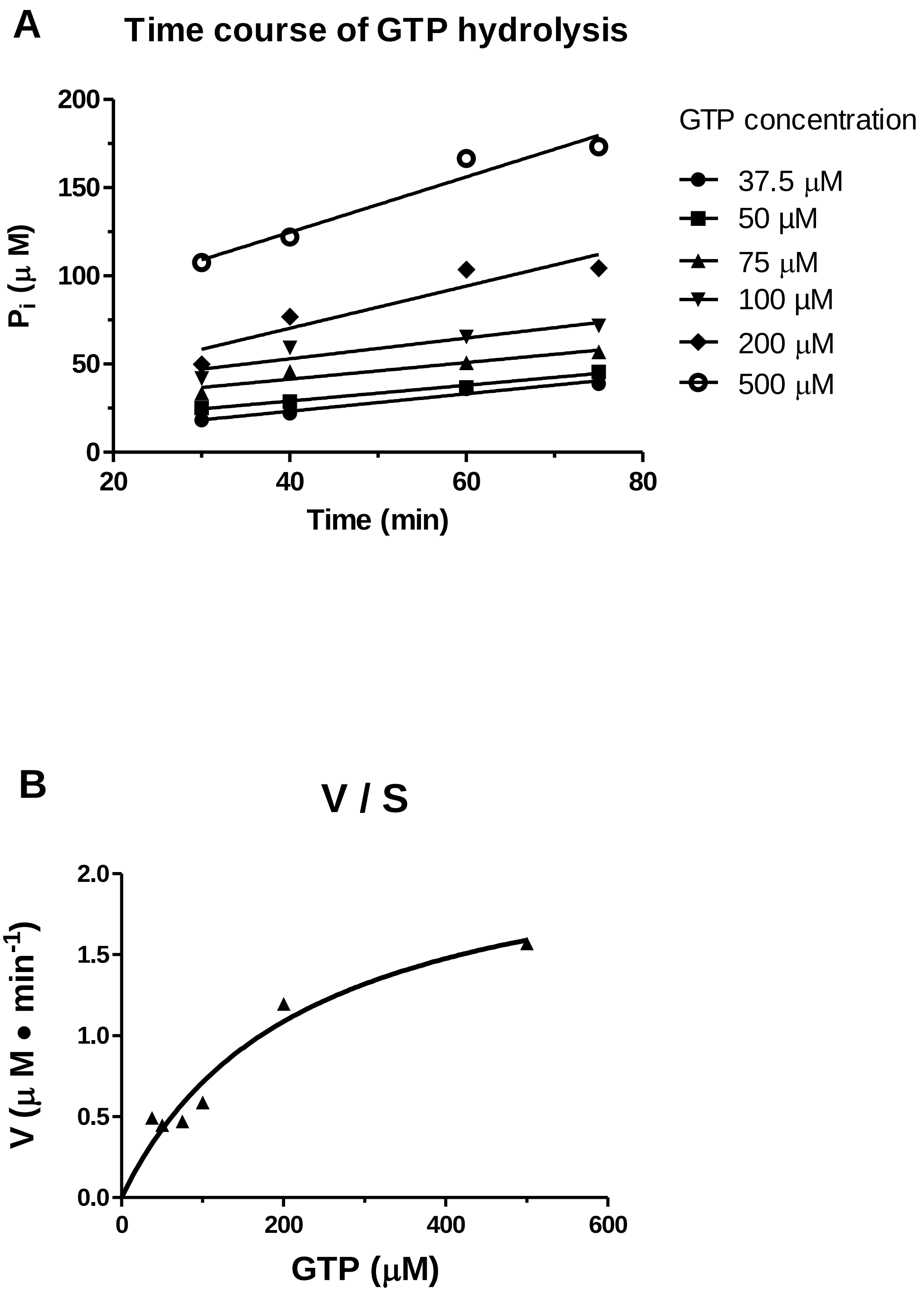

S4 Fig. A typical experimental determination of kinetic parameters for RdRp activity of NS5. A) An example of the time course for GTP hydrolysis to determine steady state kinetics. B) The enzyme rates (slopes) from the time course plots versus GTP concentrations. This is a V versus S plot with the kinetic parameter values generated by non-linear regression of this secondary plot. The plots were generated by GraphPad Prism version 5.04.
