# Supplementary material for: Glutathionylation of dengue and Zika NS5 proteins affects guanylyltransferase and RNA dependent RNA polymerase activities
Source: PLoS One. 2018 Feb 22;13(2):e0193133. doi: 10.1371/journal.pone.0193133 (PMC5823458; doi:10.1371/journal.pone.0193133)
Supplement: S1 Table — The glutathionylated cysteine is shown in red with the residue number given in superscript. LC/MS/MS was performed by Dr. Sze Siu Kwan at the NTU Mass Spec core facility at the School of Biological Sciences, Nanyang Technological University, Singapore. (DOCX) [file pone.0193133.s005.docx]

| **Peptide sequence** | **Protein domain** |
| --- | --- |
| MAISGDDC^665^VVKPLDDRFASALTALNDMGKIR | C-terminus |
| DLRLAANAIC^780^SAVPSHWVPTSR | C-terminus |
| EDQWC^847^GSLIGLTSR | C-terminus |

**S1 Table. Dengue NS5 peptides identified by mass spectrometry as containing the glutathionylated cysteine.**

The glutathionylated cysteine is shown in red with the residue number given in superscript. LC/MS/MS was performed by Dr. Sze Siu Kwan at the NTU Mass Spec core facility at the School of Biological Sciences, Nanyang Technological University, Singapore.
